# Primary Hyperoxaluria Type 1: Clinical, Paraclinical, and Evolutionary Aspects in Adults from One Nephrology Center

**DOI:** 10.1155/2023/2874414

**Published:** 2023-07-19

**Authors:** Hajji Meriam, Asma Bettaieb, Hayet Kaaroud, Fethi Ben Hamida, Taher Gargeh, Ridha Mrad, Kahena Bouzid, Ezzeddine Abderrahim

**Affiliations:** ^1^Department of Medicine A, Charles Nicolle Hospital, Tunis, Tunisia; ^2^Kidney Pathology Laboratory LR00SP01, Charles Nicolle Hospital, Tunis, Tunisia; ^3^Faculty of Medicine of Tunis, El Manar University, Tunis, Tunisia; ^4^Department of Nephrology, Mongi Slim Hospital, La Marsa, Tunis, Tunisia; ^5^Department of Pediatrics, Charles Nicolle Hospital, Tunis, Tunisia; ^6^Department of Genetics, Charles Nicolle Hospital, Tunis, Tunisia; ^7^Department of Biochemistry, Charles Nicolle Hospital, Tunis, Tunisia

## Abstract

**Introduction:**

Primary hyperoxaluria type 1 (PH1) is a rare and inherited condition of urolithiasis. The aim of our study was to analyze clinical, paraclinical, and evolutionary aspects of PH1 in adult patients in our Nephrology department.

**Methods:**

We conducted a retrospective single-center study between 1990 and 2021. We collected patients followed for PH1 confirmed by genetic study and/or histopathological features of renal biopsy and morphoconstitutional analysis of the calculi.

**Results:**

There were 25 patients with a gender ratio of 1.78. The median age at onset of symptoms was 18 years. A delay in diagnosis more than 10 years was noted in 13 cases. The genetic study found the I244T mutation in 17 cases and 33-34 InsC in 4 cases. A kidney biopsy was performed in 5 cases, on a native kidney in 4 cases and on a graft biopsy in one case. The analysis of calculi was done in 10 cases showing type Ic in 2 cases. After a median follow-up of 13 years (1 year–42 years), 14 patients progressed to end-stage chronic renal failure (ESRD). The univariate study demonstrated a remarkable association with progression to ESRD in our population (44% vs. 56%) RR = 13.32 (adjusted ORs (95% CI): 2.82–62.79) (*p* < 0.01).

**Conclusion:**

Progression to ESRD was frequent in our series. Early diagnosis and adequate management can delay such an evolution.

## 1. Introduction

Primary hyperoxaluria type 1 (PH1) is a rare and severe inherited disease affecting glyoxylate and oxalate metabolism [1, 2]. It is an autosomal recessive (AR) disorder, classified into 3 types depending on the mutated protein. The clinical presentation is dominated by oxalocalcic urinary lithiasis and nephrocalcinosis.

In Tunisia, PH1 is responsible for 13% of end-stage renal disease (ESRD) in children compared to 0.3% in Europe and 0.7% in North America [3, 4]. The aim of our study was to assess the different clinical, paraclinical, and evolutionary aspects of this disease.

## 2. Methods

### 2.1. Study Design

This was a retrospective and descriptive study conducted in our department over a 31-year period (1990–2021). From a series of 73 patients followed up for hereditary urinary lithiasis (HUL) of all causes, we collected 25 patients diagnosed with PH1. We documented those who were followed up in consultations or hospitalized for PH1.

### 2.2. Inclusion Criteria

Inclusion criteria included patients who have been diagnosed with PH1, that is, confirmed by genetic study, renal histology, or the presence of a urinary stone type Ic on morphoconstitutional analysis.

### 2.3. Participants

The patients were referred to our department for 3 reasons: firstly, for an etiological assessment of a urinary lithiasis in 12 cases; secondly, for a follow-up in the adult age from the pediatrics department in 10 cases; and finally, for an exploratory investigation of a renal failure in 3 cases. Oxaluria was measured in 11 of our patients. Concentration hyperoxaluria was defined as a concentration >0.3 mmol/l and flow hyperoxaluria as a flow rate ≥0.45 mmol/24 h or a urine oxalate to creatinine ratio (Ox/Creat) >0.03. For the genetic study, only AGXT mutations were investigated.

### 2.4. Definitions

Glomerular filtration rate (GFR) was estimated by the MDRD formula for adults and the Shwartz formula for children. Chronic kidney disease (CKD) stages were determined according to the KDIGO recommendations [5].

The urinary stones analyzed were classified according to the morphoconstitutional classification of Michel Daudon [6].

### 2.5. Statistical Analysis

Data were entered and analyzed using Excel 365 and SPSS 20.0 software. We calculated the mean annual decline in glomerular filtration rate by dividing the difference in the sum of glomerular filtration rates at the start of follow-up and at the last visit by the total follow-up time in years. We compared survival curves (using the Kaplan–Meier method) based on the log rank test and performed logistic regression using the Cox proportional hazards model. The parameters studied were age, gender, family history of CKD, time to etiological diagnosis, recurrence, aetiologies of HUL, occurrence of urinary tract infections or acute renal failure, stage of CKD at diagnosis, bilaterality of stone, presence of coralliform stone or nephrocalcinosis, and urological treatment.

We calculated incidence rates of CKD by relating the number of patients who started renal replacement therapy to the duration of follow-up. Confidence intervals were calculated at 95% using standard formulae. In all cases, the test was considered significant when the *p* level of significance was below 0.05.

## 3. Results

There were 16 males and 9 females with a gender ratio of 1.78. The age of onset of lithiasis ranged from 2 to 55 years with a median of 18 years. The median time to etiological diagnosis was 10 years (range: 0–39 years). A delay in diagnosis of more than 10 years was noted in 13 cases (52%). The presence of parental consanguinity was specified in 22 cases. It was found in 20 cases (91%). It was of first, third, and second degree in 13, 5, and 1 cases, respectively. One case was distantly related. A family history of urinary lithiasis was found in 17 cases (68%).

We identified 4 clinical forms: the child and adolescent form with progressive alteration of renal function was noted in 10 of our patients, the adult form was observed in 13 of our patients, and recurrence after renal transplantation without a precise diagnosis before transplantation was noted in one case. One patient was found to have a family survey following in the presence of an index case. The circumstances of disease discovery were specified in 24 cases (Table 1). The most common presenting symptom was renal colic.

Oxaluria was measured in 11 cases, with a mean oxaluria of 0.484 mmol/l (range: 0.238–0.953 mmol/l). We noted flow hyperoxaluria in all cases and concentration hyperoxaluria in 8 cases (73%). Twelve patients had normal renal function (48%). Thirteen had CKD (52%). One patient was at stage 3*A*, one was at stage 3*B*, and three were at CKD stage 4. Seven patients had end-stage CKD from the beginning. One patient had unclassified CKD due to lack of height data at the age of 8 years. Ten patients had a stone analysis which was type Ia in 8 cases (80%) and Ic in 2 cases (20%). An additional infection lithiasis was noted in one case (10%). Crystalluria was positive in 12 cases. Whewellites was noted in 11 cases (92%) and weddellites in one case (8%).

**Figure 1:**
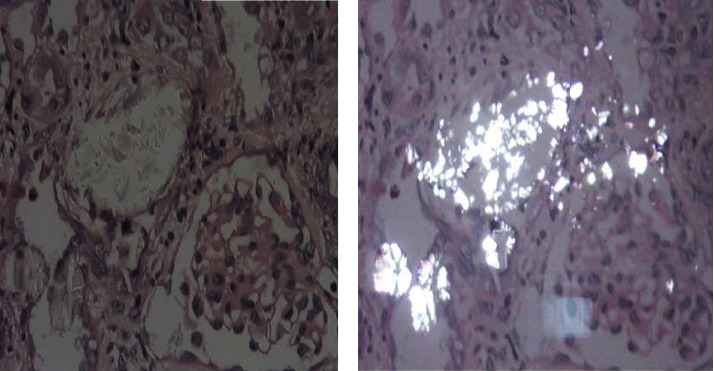
(a) Abundant precipitation of calcium oxalate crystals in the tubular lumens in a patient of our series. (b) Refringence of calcium oxalate crystals in polarised light. Haematoxylin-eosin in polarised light ×40.

On a kidney scan, urinary lithiasis was bilateral in 18 cases. Nephrocalcinosis was found in 7 cases, associated with urinary stones in 6 cases and isolated in one case. A coralliform stone was noted in 2 cases. Genetic study revealed the I244T mutation in 17 cases and the 33-34 InsC mutation in 4 cases. Renal biopsy was performed in 5 cases (6%). Four cases were oxalosis in native kidneys, one of which was postmortem, and one case was an unrecognized oxalosis diagnosed on graft biopsy.  Figure 1 shows the histological appearance of renal oxalosis in the native kidney. Overall, PH1 diagnosis was confirmed by genetic study in 21 cases, by renal histology alone in 2 cases, and by morphological analysis of the stone which was type Ic in 2 cases (8%).

Medical treatment included a diuresis of 3 l/m^2^/24 h and a low oxalate diet for all our patients and vitamin B6 for 13 of our patients. After a median follow-up of 13 years (extremes: 1 year–42 years), 14 patients progressed to end-stage CKD with recourse to renal replacement therapy at a mean age of 36 years (extremes 18–54 years). Two patients had an isolated kidney transplant (RT). The first patient was transplanted at the age of 42 years from a brain-dead donor. The course was marked by the occurrence of acute renal failure at D2 post-RT, nonreversible in relation to unrecognized oxalosis which was diagnosed on graft biopsy. The second patient had an isolated RT after 7 years of extrarenal purification. She had PH1 related to a homozygous mutation of the I244T allele. Clinically, she had no organomegaly. Blood oxalinity was normal at 29, 13, and 31 *μ*mol/l. She had no osteocondensing lesions on imaging and no oxalic deposits on fundus and osteoarticular biopsy. She was transplanted from a related living donor (her mother) carrying the same mutation in the heterozygous state. The evolution was favourable after hyperhydration and vitamin B6 supplementation. She retained normal renal function after 7 years of transplantation.

A family screening was performed in 6 families, five by genetic study and one by urine oxaluria determination. It led to a positive diagnosis of unrecognized urinary lithiasis in 4 families, with the number of patients screened varying between 1 and 3 per family.

The univariate study demonstrated a remarkable association with progression to ESRD in our population (44% vs. 56%) RR = 13.32 (adjusted ORs (95% CI): 2.82–62.79) (*p* < 0.01).

The incidence of CKD was high among our PH1 patients (*n* = 8/18; 3.42%) (adjusted ORs (95% CI): 1.05–5.79) compared to other patients with other hereditary urolithiasis in our department, but the difference was not statistically significant. Long-term renal survival in PH1 was observed to be 33% at 30 years.

## 4. Discussion

PH1 is predominantly a disease of young children, but the age range is from birth to over 60 years [2]. In our series, the onset of symptomatology was in adulthood in 13 cases (52%). This is explained by a selection bias as it is a recruitment of patients in an adult nephrology department. The gender ratio varies according to the series, with a male predominance noted in the series by Soliman et al. [7] and Gargah et al. [8] with a gender ratio of 1.36 and 1.2, respectively, while a female predominance was noted in the series by Nagara et al. with a gender ratio of 0.63 [9]. In our study, we noted a male predominance with a gender ratio of 1.78.

PH1 presents in 5 clinical forms [10]. The first form, known as infantile, is severe, with rapid progression to CKD as a result of high hepatic oxalate production. These patients are managed in paediatrics. The second form is that of children and adolescents with progressive alteration of renal function. This form was noted in 10 of our patients. In the series by Gargah et al. [8], the most frequent initial symptoms of childhood were hyperuremia in 44% of cases, urinary tract infections in 26% of cases, and abdominal or back pain in 20% of cases. The third form, that of the adult, was observed in 13 of our patients. The fourth form is the recurrence after renal transplantation without precise diagnosis before the transplantation which was noted in one of our cases. The fifth form is that of subjects identified following family screening in the presence of an index case. This form was observed in one of our cases. In the series by Gargah et al. [8], a discovery in the context of a family investigation was noted in 9% of cases.

PH1 is classified into 3 types depending on the mutated protein. Type 1 is the most common and is due to a mutation in the AGXT gene coding for alanine glyoxylate aminotransferase (AGT).

In Tunisia, only the search for specific mutations in PH1 is done [11]. The AGXT mutations identified differ according to geographical origin [3, 9, 12, 13]. The so-called “Maghrebian” I244T mutation is predominant in all Tunisian regions. It has been described in Morocco, Algeria, Libya, Turkey, Pakistan, and the Canary Islands [14]. It was also the most frequent mutation in our series.

There is prevalence of consanguineous marriages and geographic endogamy in Tunisia, mainly in the rural areas. Consanguinity rate ranges from 20.1% to 39.33% [15, 16].

Due to the high occurrence of consanguineous unions, a previous study indicated a six-fold rise in the risk of AR diseases [17]. A retrospective analysis involving 425 Tunisian patients with AR disorders discovered consanguinity in 69.4% of the cases, with first cousin marriages being prevalent at 48.94% [18]. In specific southern regions, the rate of consanguinity rose to 65.26%. The majority of mutations were observed in the homozygous state. Furthermore, the study noted that geographic endogamy was present in 93.92% of the cases investigated. The authors estimated a seven-fold increase in the likelihood of AR diseases due to consanguinity, with certain cases having a risk as high as 24-fold.

The InsC 33-34 mutation was identified in Kasserine, Mahdia, and Sfax, which are towns located in the central region of Tunisia, and was found in 4 of our cases. It has been described previously in Italy [3, 9, 12, 13]. The only known aspect of the genotype-phenotype relationship in PH1 is the response to pyridoxine in patients with Gly170Arg and Phe152Ile mutations, which may improve renal prognosis [10].

CKD at the time of the etiological diagnosis of urinary lithiasis was noted in 13 cases (52%), 7 of which were at stage 5 of CKD. This is explained by the severity of the disease and the delay in diagnosis in our patients. The frequency of type Ia in our study is probably explained by a delay in diagnosis which causes a change in the morphology of type Ic.

In our series, a renal biopsy was performed in 5 cases. Four of these cases were oxalosis on native kidneys, one of which was postmortem, and one on graft biopsy. The positive diagnosis was made on renal histology in 5 cases in the series by Soliman et al., 4 on native kidneys and one on renal graft [7], and in 9 cases in the series by Gargah et al. [8]. Renal biopsy found crystals of monohydrated calcium oxalate, arranged in a rosette or needle-like radial pattern. These crystals are mainly located in the tubular lumen. They are birefringent in polarised light (Figure 1). The massive nature of the calcium oxalate deposits points to the primary origin of POH but does not allow its classification (Figure 1).

The aim of treatment is to reduce calcium oxalate supersaturation and to delay progression to advanced renal failure and systemic oxalosis [19, 20]. It involves a diuresis treatment of 3 l/m^2^/24 h regularly distributed throughout the day, especially at bedtime, to avoid nocturnal supersaturation, a diet low in oxalate and a crystallisation inhibitor (potassium citrate and magnesium salts). Pyridoxine (vitamin B6), a cofactor of AGT, is beneficial in patients with PH1 with residual enzyme activity by diverting oxalate metabolism to the more soluble glycocoll. It can reduce oxaluria by up to 30% in 30% of patients [20–22]. Vitamin B6 was prescribed for 13 of our patients. Several promising treatments are under investigation such as Oxalobacter formigenes, Lumasiran, Stirpental, gene therapy, and hepatocyte transplantation [1, 2].

Combined liver-renal transplantation is the best strategy for CKD. Isolated preemptive liver transplantation can be proposed before stage 4 CKD but poses ethical problems, whereas isolated renal transplantation has a high risk of graft recurrence [2, 10, 11, 23]. In our series, one patient presented with an early recurrence of PH1 without a definite diagnosis before transplantation. The second patient had an isolated kidney transplant from a related living donor. The evolution was favourable with preventive measures combining hyperhydration and vitamin B6. She retains normal renal function after 7 years of transplantation.

CKD was common in our series at diagnosis and during follow-up. In an American study of primary hyperoxaluria, the annual decline in eGFR differed according to the stage of CKD. It was 2.3, 5.3, 14.7, and 16.6 ml/min for stages 2, 3*A*, 3*B*, and 4, respectively [24]. It has been reported that type 1 primary hyperoxaluria has the worst renal prognosis with a renal survival of 27% at 30 years for type 1 primary hyperoxaluria, compared with 92% and 95% for types 2 and 3, respectively [25]. It is explained by the massive deposition of calcium oxalate crystals in the renal interstitium where they induce an inflammatory response and progressive interstitial fibrosis [11, 24, 26].

Our series is one of the few Tunisian and international series dealing with adult-onset PH1 with a good follow-up. However, the main limitations of the study were the retrospective character which confronted us with a number of missing data and patients lost to follow-up and the genetic study analyzing only the mutations of the alanine AGXT gene.

## 5. Conclusion

Our study sheds light on PH1 as a rare and an underdiagnosed condition. The most frequent genetic mutation in our series was I244T. Our findings reveal a poor renal prognosis for PH1, with more than half of our patient's experiencing progression to CKD. Timely diagnosis and effective management strategies are crucial in delaying the progression of PH1.

## Figures and Tables

**Table 1:** Circumstances of discovery of primary hyperoxaluria.

Circumstances of diagnosis *N* (%)	*N* (%)
Nephritic colic	13 (54)
NC and gross haematuria	2 (8)
Lower back pain	1 (4)
Chronic kidney disease	1 (4)
Hypertension	2 (8)
Postsurgical renal failure	2 (8)
Acute bladder retention	1 (4)
Polyuric polydipsic syndrome	1 (4)

## Data Availability

The data used to support the findings of this study are available from the corresponding author upon request.
